# Time, location and frequency of snack consumption in different age groups of Canadians

**DOI:** 10.1186/s12937-020-00600-5

**Published:** 2020-08-15

**Authors:** Hassan Vatanparast, Naorin Islam, Hedyeh Masoodi, Mojtaba Shafiee, Rashmi Prakash Patil, Jessica Smith, Susan J. Whiting

**Affiliations:** 1 College of Pharmacy and Nutrition, University of Saskatchewan, Saskatoon, SK S7N 4Z2 Canada; 2 School of Public Health, University of Saskatchewan, Saskatoon, SK S7N 4Z2 Canada; 3 Catholic San Antonio University, Murcia, Spain; 4 Bell Institute of Health and Nutrition, General Mills, Minneapolis, MN 55427-3870 USA

**Keywords:** Snack, Time, Location, Frequency, National Survey, Canadian population

## Abstract

**Background:**

The location and time of snack consumption may influence the composition, nutrient content and portion sizes of snacks. In this study, we aimed to determine and compare the time, location and frequency of snack consumption among different age groups of Canadians.

**Methods:**

Nationally representative dietary data from the 2015 Canadian Community Health Survey (CCHS) were used (19,677 participants aged ≥2 years). Dietary data were obtained using 24-h dietary recalls. Participants were categorized according to the frequency of snack consumption (1 time, 2–3 times, ≥4 times). The snack consumption over 24 h was divided into four time periods: before 10 am, 10 am to 3 pm, 3 pm to 8 pm and 8 pm to 12 am. Meal and snack location was categorized as at home; someone else’s home; restaurants; and other.

**Results:**

Snacking 2–3 times per day was the most common reported frequency (53.0%). Snacking at home (73.0%) was more prevalent than snacking away from home (27.0%). The most frequently reported time for snacking among Canadians was 3 pm to 8 pm (36.3%), and the least frequently reported time was before 10 am (8.1%). Snacking contributed to a high proportion of Milk and Alternatives (23.7%) and Vegetables and Fruit (23.4%) food groups and lesser proportions of Grain Products (15.4%) and Meat & Alternatives (9.4%) food groups among Canadians.

**Conclusions:**

Home is the main location, 3 pm to 8 pm is the main time, and 2–3 times per day is the most common reported frequency for snacking. A full understanding of snacking behaviors is needed in order to develop targeted strategies to improve the quality of snack food choices.

## Background

Snacking is a prominent dietary habit in most parts of the world and has increased over time in prevalence, frequency, portion size, and energy contribution, especially among high-income countries (HICs) [1–3]. In the United States, for example, the prevalence of snack consumption (from 71 to 97%), the number of snacks consumed per day (from 1.26 to 2.23), and the percentage of daily energy provided by snacks (from 18 to 24%) dramatically increased among adults from 1977 to 2003–2006 [1]. In Canada, as we recently showed [4], snacking is a popular eating behavior, with more than 80% of Canadians having at least one snacking episode per day. Snacking is also a major source of daily energy intake among Canadian population, providing near one-quarter of total energy intake [4].

The location and time of snack consumption may influence the composition, nutrient content and portion sizes of snacks [5–7]. Data on dietary behaviors of the US adults revealed that eating at fast food/convenience and sit-down restaurants were associated with lower odds of eating healthy snacks compared to eating at home [5]. It has been estimated that each snack away from home adds more than 100 cal to the daily intake of US adults [6]. Further, a study conducted in Northern Ireland showed that the portion sizes of away-from-home snacks of children were generally higher than snacks consumed at home [7]. In addition, the morning snack period was found to be the most nutrient-dense snacking period among children in the United States [8]. Therefore, to develop targeted strategies to improve the quality of snack choices of Canadians, it is essential to increase awareness of snacking behavior and understanding of time and place of snack consumption.

To our knowledge, no previous studies have determined the time and location of snack consumption among Canadians. Therefore, using nationally representative nutrition data from the 2015 Canadian Community Health Survey (CCHS), our main objective was to determine and compare the time of day and location (at home vs away-from-home) of snack consumption among Canadians by age group. The secondary objective was to determine the percentage contribution of snacking to intake of food groups, using the 2007 Canada’s Food Guide.

## Methods

We used nutrition data from the 2015 CCHS, which is a cross-sectional population-based survey conducted in the Canadian population aged 2 years and over living in the 10 Canadian provinces. The CCHS 2015 Nutrition survey was carried out on a population of 20,487 participants using complex sampling methods by Statistics Canada. The sample is representative at national and provincial levels. Details of sampling strategy are explained elsewhere [9, 10]. Due to missing data or anticipated differences in eating habits, we excluded children less than 2 years, pregnant or breastfeeding women, and individuals with missing/invalid dietary recalls. In addition, those who were defined as “snack non-consumers” (i.e. those individuals that did not report any snack eating occasions on day 1 of 24-h recall), were not included in our analysis [4]. Our final sample size was 16,179.

As described elsewhere [4], a computer-assisted dietary recall method known as the Automated Multiple Pass Method (AMPM) was used to provide details about participants’ food and beverage consumption including eating occasion (e.g., breakfast, lunch, dinner, and snack), location of consumption (e.g., home, school, and work), time of consumption and types and amounts of foods consumed [11]. For children aged 1–6 years, data were obtained by proxy interviews and those aged 6 to 12 years old participated in the survey with parental guidance. A non-proxy method was used to interview individuals aged 12 years and older [9]. All data analyses were performed at the Sky Research Data Center, University of Saskatchewan. The vetting process of Statistics Canada was followed in order to release the results of the study.

In this survey, all the participants self-reported the type of eating occasion on their 24-h dietary recall. Individuals who reported consuming any snack on day 1 of the 24-h dietary recall were defined as “snack consumers”. As reported earlier [4], out of all participants, 16,179 individuals reported snack consumption. Participants were categorized according to the frequency of their snack consumption per day (1 time, 2–3 times, ≥4 times). The snack consumption over 24 h was divided into four time periods for this analysis: before 10 am, 10 am to 3 pm, 3 pm to 8 pm and 8 pm to 12 am. Meal and snack location was categorized as at home; someone else’s home; restaurants; and other. Examples of other locations include work, school, child care center, adult care center, cafeteria, sport/entertainment venue, grocery store/other store, bar/tavern/lounge, church/temple/other religious site, and car/other vehicle. The total sample was divided into five age groups: 2–5 years (younger children), 6–12 years (children), 13–18 years (adolescents), 19–54 years (adults) and ≥ 55 years (older adults).

The percentage contribution of snacks to Canada’s Food Guide food groups and subgroups was calculated for the five age groups. Canada’s Food Guide 2007 [12] was used to define five main food groups (Vegetables and Fruit, Milk and Alternatives, Meat and Alternatives, Grain products, and Other Foods and Beverages) and their subgroups.

Data are presented as percentages and standard errors (SE). To produce the population-level estimates, appropriate weighting and bootstrapping procedures were applied as per Statistics Canada’s recommendations [10]. The comparisons were made by confidence interval overlapping technique. All data analyses and statistical processes were conducted using SAS, version 9.4 (SAS Institute).

## Results

Overall, 80.1% of Canadian males and 80.8% of Canadian females reported consuming at least one snack per day in 2015. Thirty-seven percent of snack consumers reported only one snacking episode per day and the remaining 63% reported two or more snacking episodes per day (data not shown). Snacking 2–3 times per day was the most common reported frequency (53.0%). The proportion of Canadian children (2–18 years) and adults (≥19 years) among different frequencies of snack consumption is reported in Table 1. Among both males and females, the proportion of children significantly increased from 14.5 and 15.1%, respectively, in one-time snack consumers to 30.5 and 36.2% in those consuming four or more snacks per day. Conversely, the proportion of male and female adults was significantly lower in higher frequencies of snack consumption (69.5 and 63.8%, respectively) compared to lower frequencies (85.5 and 84.9%, respectively).

**Table 1:** The proportion of Canadian children and adult snack consumers among different snack consumption frequencies

Frequency	Males				Females			
	Children (*n* = 2,927,629)		Adults (*n =* 10,429,887)		Children (*n =* 2,890,187)		Adults (*n =* 10,649,097)	
	Mean	SE	Mean	SE	Mean	SE	Mean	SE
**1 time**	14.5 ^**a,b**^	0.8	85.5 ^**a,b**^	0.8	15.1 ^**a,b**^	0.9	84.9 ^**a,b**^	0.9
**2–3 times**	25.7	0.9	74.3	0.9	22.5 ^**c**^	0.8	77.5 ^**c**^	0.8
**≥4 times**	30.5	2.9	69.5	2.9	36.2	2.3	63.8	2.3

Values are presented as mean ± SE. Significant differences measured by confidence interval overlapping technique, a: 1 time vs 2–3 times; b: 1 time vs ≥4 times; c: 2–3 times vs ≥4 times

**Fig. 1:**
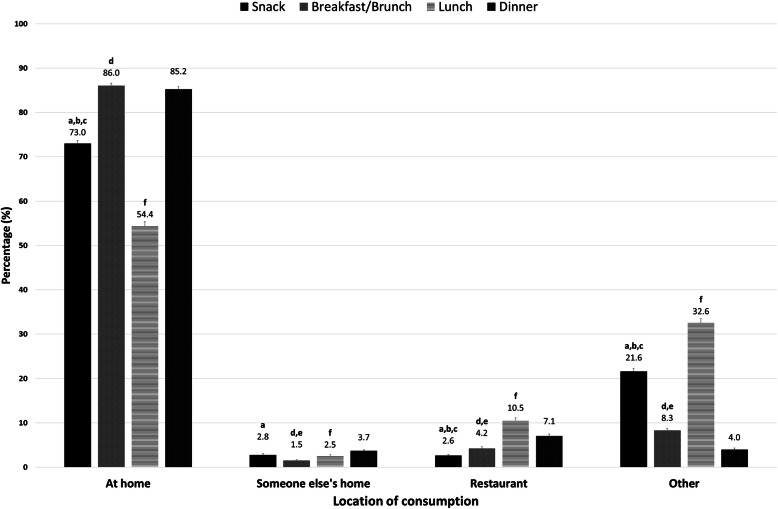
Location of meal and snack consumption among Canadians. Significant differences measured by confidence interval overlapping technique, a: Snack vs Breakfast/Brunch; b: Snack vs Lunch; c: Snack vs Dinner; d: Breakfast/Brunch vs Lunch; e: Breakfast/Brunch vs Dinner; f: Lunch vs Dinner. Total population: *N* = 26,896,800

The location of meal and snack consumption among Canadians is shown in Fig. 1. The home was the primary location for meal (breakfast, lunch, and dinner) and snack occasions. Overall, 73.0% of Canadians reported having their snack at home, which was significantly lower than the corresponding values of 86.0 and 85.2% for breakfast and dinner, respectively. The percentage of Canadians having lunch at home was significantly lower than the other three eating occasions (54.4%). Only a small percentage of Canadians reported having a snack at other peoples’ homes (e.g. the home of friends, relatives and childminders) (2.8%) and in restaurants (2.6%). Eating snacks at other places, such as child care centers, adult care centers, school and work was common, with more than 21% of Canadians reporting “other” locations for snacking. The rate of having a meal in the restaurant was highest for lunch (10.5%), followed by dinner (7.1%) and breakfast (4.2%). Dinner was the most (3.7%) commonly consumed meal at other peoples’ homes, while lunch was the most common meal eaten at other locations (32.6%, Fig. 1).

**Fig. 2:**
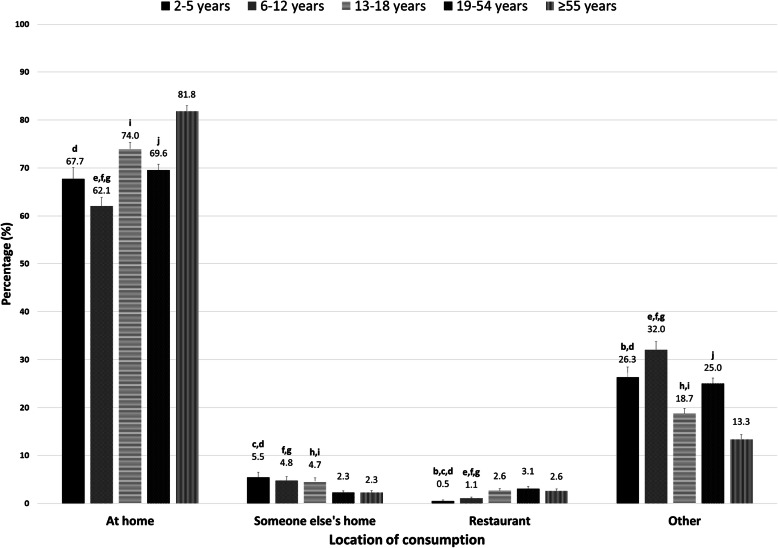
Location of snack consumption among different age groups. Significant differences measured by confidence interval overlapping technique, **a**: 2–5 years vs 6–12 years; **b**: 2–5 years vs 13–18 years; **c**: 2–5 years vs 19–54 years; **d**: 2–5 years vs ≥55 years; **e**: 6–12 years vs 13–18 years; **f**: 6–12 years vs 19–54 years; **g**: 6–12 years vs ≥55 years; **h**: 13–18 years vs 19–54 years; **i**: 13–18 years vs ≥55 years; **j**: 19–54 years vs ≥55 years. 2–5 years: *n* = 1,181,823, 6–12 years: *n* = 2,407,637, 13–18 years: *n* = 2,228,356, 19–54 years: *n* = 12,768,195, ≥55 years: *n* = 8,310,789

The location of snack consumption among different age groups is illustrated in Fig. 2. Home was the most frequently reported location for the consumption of snacks for all age groups. The lowest rate of having a snack at home was observed in children, especially those aged 6–12 years (62.1%). Near 82% of older adults (≥55 years) reported having snack at home, which was significantly higher than the other age groups. The rate of having a snack at other peoples’ homes was significantly higher in children and adolescents than in adults aged ≥19 years. Conversely, the rate of having a snack in the restaurant was significantly higher in adolescents and adults than in children. The highest rate of having a snack at “other” locations was found in children, especially those aged 6–12 years (32.0%). Only 13.3% of older adults (≥55 years) reported having a snack at “other” locations, which was significantly lower than the other age groups (Fig. 2).

**Fig. 3:**
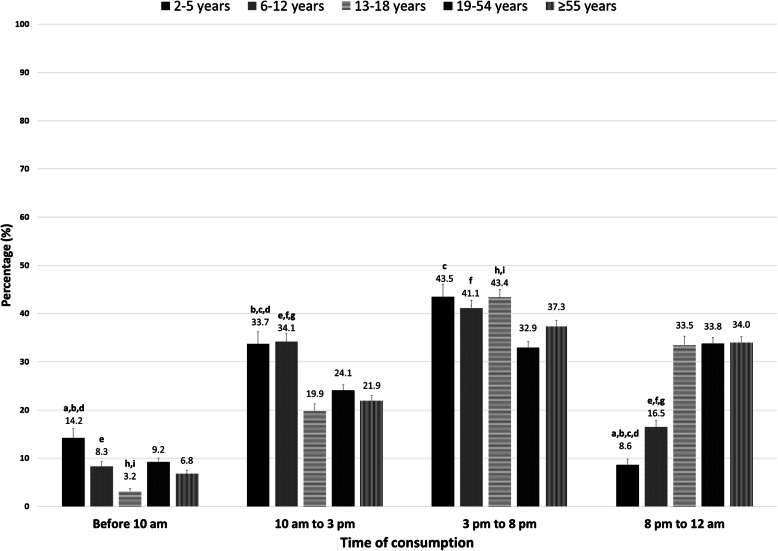
Time of snack consumption among different age groups. Significant differences measured by confidence interval overlapping technique, **a**: 2–5 years vs 6–12 years; **b**: 2–5 years vs 13–18 years; **c**: 2–5 years vs 19–54 years; **d**: 2–5 years vs ≥55 years; **e**: 6–12 years vs 13–18 years; **f**: 6–12 years vs 19–54 years; **g**: 6–12 years vs ≥55 years; **h**: 13–18 years vs 19–54 years; **i**: 13–18 years vs ≥55 years; **j**: 19–54 years vs ≥55 years. 2–5 years: *n* = 1,181,823, 6–12 years: *n* = 2,407,637, 13–18 years: *n* = 2,228,356, 19–54 years: *n* = 12,768,195, ≥55 years: *n* = 8,310,789

The time of snack consumption among different age groups is shown in Fig. 3. During the 24-h period, the most frequently reported time for snacking was 3 pm to 8 pm in all age groups, except adults aged 19–54 years. Only 3.2% of adolescents reported having a snack before 10 am, which was significantly lower than the other age groups (Fig. 2). The highest rate of having a snack before 10 am was observed in children aged 2–5 years (14.2%). The rate of snacking between 10 am and 3 pm was significantly higher in children (33.7% in children aged 2–5 years and 34.1% in children aged 6–12 years) than in adolescents and adults. Adults aged 19–54 years reported the lowest rate of having a snack between 3 pm and 8 pm among different age groups (32.9%). At last, only 8.6% of children aged 2–5 years and 16.5% of children aged 6–12 years reported having a snack between 8 pm and 12 am, which was significantly lower than the other age groups (Fig. 3).

The percentage contribution of snacks to Canada’s Food Guide food groups and food subgroups (servings/day) among different age groups is reported in Table 2. The percentage contribution of snack to the Vegetables and Fruit food group was significantly higher among children (31.4–33.8%) and adolescents (27.9%) than adults (22.4%) and older adults (19.9%). In terms of subgroups, adults and older adults had the lowest percentage contribution of snack to whole fruit (52.8 and 47.2%, respectively), fruit juice (12.0 and 13.5%, respectively) and vegetables (9.1 and 5.0%, respectively). Children aged 6–12 years had the highest percentage contribution of snack to whole fruit (59.3%) and fruit juice (24.7%), while the highest percentage contribution of snack to vegetables (14.0%) was observed among adolescents. There were no significant differences in the percentage contribution of snack to Grain Products food group between different age groups (Table 2). Concerning subgroups, the highest (18.9%) and the lowest (11.4%) percentage of whole grains coming from snacks were observed in adolescents and older adults, respectively. Similarly, the percent contribution of snack to non-whole grain, not enriched grain products was highest among adolescents (32.6%) and lowest among older adults (22.0%). More than 29% of Milk & Alternatives food group in children aged 2–5 years came from snacking. No significant differences were observed in percent contribution of snacks to fluid milk and fortified soy-based beverages between different age groups. The percent contribution of snacks to other milk alternatives (e.g. cheese, yogurt) was significantly higher among children (38.1–42.9%) than other age groups. The percent contribution of snack to Meat & Alternatives food group was highest among adults (10.4%) and lowest among children aged 6–12 years (6.4%). In addition, about 24% of Other Foods and Beverages such as alcoholic beverages, higher calorie beverages (e.g. energy drinks, blended coffee drinks, soft drinks, and juice drinks), lower calorie beverages (e.g. coffee, plant-based milks, diet soft drinks, diet energy drinks), high fat and/or sugar foods (e.g. desserts, candies, ice cream, coffee whitener, dips, cream, bacon, coconut, cured meats, some dried fruits, cream soups, gravies and sauces), saturated or trans fats and oils (e.g. butter, animal fats, salad dressings, oils), and cooking ingredients (e.g. dry baby food, cake mixes, instant coffee and tea, flours, leavening agents, drink mixes, protein powders, soy sauce, soup mixes, sauce mixes, spices and herbs, dessert mixes) in children aged 6–12 years and adolescents came from snacks (Table 2).

**Table 2:** The percentage contribution of snacks to Canada’s Food Guide food groups and food subgroups (servings) among different age groups

		Age groups											
		2–5 years		6–12 years		13–18 years		19–54 years		≥55 years		Total population	
		Mean	SE	Mean	SE	Mean	SE	Mean	SE	Mean	SE	Mean	SE
**Food groups and subgroups**	**Vegetables & Fruit**	33.8 ^**b,c,d**^	1.6	31.4 ^**f,g**^	1.1	27.9 ^**h,i**^	1.0	22.4	0.8	19.9	0.7	23.4	0.4
	Fruit, other than juice	58.8 ^**d**^	2.2	59.3 ^**f,g**^	1.7	56.5 i	1.8	52.8 ^**j**^	1.5	47.2	1.3	52.3	0.8
	Fruit, juice	19.1	2.7	24.7 ^**e,f,g**^	2.5	13.7	1.6	12.0	1.8	13.5	1.8	15.1	0.9
	Vegetables, all types	9.0 ^**b,d**^	1.2	11.8 ^**g**^	0.9	14.0 ^**h,i**^	0.9	9.1 ^**j**^	0.4	5.0	0.4	8.5	0.4
	**Grain Products**	15.0	0.9	17.0	0.8	17.6	0.7	15.4	0.6	14.5	0.7	15.4	0.4
	Grain products, whole grain	12.2	1.7	15.3	1.5	18.9 ^**i**^	2.0	14.7	1.3	11.4	1.3	13.9	0.7
	Grain products, non-whole grain, enriched	16.2	1.3	17.2	0.9	16.8	0.9	16.7	0.8	17.3	0.8	16.9	0.5
	Grain products, non-whole grain, not enriched	22.4	3.1	31.1 ^**g**^	2.5	32.6 ^**i**^	2.3	25.4	1.9	22.0	1.9	25.6	1.1
	**Milk & Alternatives**	29.1 ^**b,d**^	1.8	26.0	1.0	22.9	1.1	23.7	1.1	22.3	1.0	23.7	0.6
	Fluid milk and fortified soy-based beverages	16.5	1.8	14.0	1.0	14.2	1.1	14.4	1.0	11.8	0.7	13.6	0.5
	Other milk alternatives (cheese, yogurt)	42.9 ^**b,c,d**^	2.6	38.1 ^**e,f,g**^	1.7	29.4	1.5	30.0	1.3	29.4	1.4	31.3	0.8
	**Meat & Alternatives**	8.6	1.2	6.4 ^**f,g**^	0.7	8.4	0.8	10.4	0.7	9.0	0.6	9.4	0.4
	**Other foods & beverages**	18.2 ^**a,b**^	1.5	23.8 ^**g**^	1.1	24.1 ^**i**^	1.0	20.7 ^**j**^	0.9	16.4	0.7	19.8	0.5

Significant differences measured by confidence interval overlapping technique, a: 2–5 years vs 6–12 years; b: 2–5 years vs 13–18 years; c: 2–5 years vs 19–54 years; d: 2–5 years vs ≥55 years; e: 6–12 years vs 13–18 years; f: 6–12 years vs 19–54 years; g: 6–12 years vs ≥55 years; h: 13–18 years vs 19–54 years; i: 13–18 years vs ≥55 years; j: 19–54 years vs ≥55 years. 2–5 years: *n* = 1,181,823, 6–12 years: *n* = 2,407,637, 13–18 years: *n* = 2,228,356, 19–54 years: *n* = 12,768,195, ≥55 years: *n* = 8,310,789, total population: *n* = 26,896,800

## Discussion

To our knowledge, this is the first study to determine the time and location of snack consumption among a representative sample of Canadians. Among snack consumers, snacking at home was more prevalent than snacking away from home. The most frequently reported time for snacking was 3 pm to 8 pm, and the least frequently reported time was before 10 am.

Our results showed that snacking at home was more prevalent than snacking away from home among Canadians. However, adults were more likely than children and less likely than older adults to snack at home. A study evaluating the snacking behavior among American seniors (≥ 55 years) showed that the majority of participants (89.2%) reported having a snack at home more often than any other location such as at work (6.8%), home of a friend (1.8%), or restaurant (1.4%) [13]. In another study, home was the most frequent place of snacking (58% of all snacks) among Norwegian adults aged 18–70 years, followed by work/school (23%), travel/meeting (10%), other private households (6%) and restaurant/cafe/fast-food outlet (3%) [14]. This study also reported that snacks consumed at home contained more energy than snacks consumed at work/school and contained less energy than snacks consumed at restaurants/cafe/fast-food outlets [14]. Data on dietary behaviors of 226 adults recruited from five cities in the US demonstrated that the odds of snacking, whether healthy or unhealthy, was approximately 1.5 times greater at work compared to at home. In addition, eating at sit-down restaurants and fast food/convenience store was associated with lower odds of eating healthy snacks compared to eating at home [5]. The propensity of adults to snack away from home, especially at work, highlights the opportunity for health promotion initiatives encouraging healthy food and drink availability at work.

Several studies have previously reported the number or proportion of snacks consumed at home and away from home by children and adolescents [7, 15–19]. Our results revealed that children, especially older ones (6–12 years), tended to consume more snacks away from home (about 38%) than other age groups. These findings are consistent with other publications showing the importance of snacks away from home for this age group. Kerr and colleagues reported that the frequency of snacking was significantly higher at home (2.0 snacking occasions/day) compared with that away from home (0.7 snacking occasions/day) among Northern Ireland children aged 5–8 years. However, the portion sizes of away-from-home snacks were generally higher than snacks consumed at home [7]. In a study conducted in 860 Dutch children aged 7–12 years, nearly half of the energy-dense snack food (EDSF) events took place at home, with the remaining half occurring at school (17.1%), a friend’s home (15.2%) and other locations [17]. In a nationally representative sample of children in grades one through twelve, Briefel et al. found that 83 and 40% of participants consumed at least one snack at home and school, respectively [18].

It has been shown that peer social influence has a major effect on snacking food behavior in children and adolescents [20, 21]. Furthermore, there is a significant association between student snack food purchases and school policies on types of food that can be sold [22]. Since a large proportion of snacking events in children and adolescents occurs away from home, especially in schools, educating school-aged children about healthy and safe snack foods and implementing healthy school food policies may promote healthy snacking behavior among these age groups. For instance, it has been reported that the 2005 vending machine ban in French secondary schools resulted in a 10-g reduction in sugar intake of students from morning snacks [23].

Among Canadians, the most frequently reported time for snacking was 3 pm to 8 pm, followed by 8 pm to 12 am. Adolescents were the least likely to snack before 10 am and children were the least likely to snack between 8 pm and 12 am. Similar to our findings, a study conducted in Greek adults, aged 20–50 years, reported snacking occurred mainly during the afternoon, between 5 pm and 8 pm, and the least common time of day for snacking was morning, between 5 am and 10 am [24]. Results from a cross-sectional population-based survey by Ovaskainen et al. showed that Finnish adults obtained the majority of energy from snacks during the evenings and nights [25]. Among the Chinese population, the evening was the most preferred snacking period over time, followed by afternoon [26]. Cross and colleagues reported that afternoons were the most popular time for snacking among all age groups in the United States, except for seniors who preferred an evening snack. Moreover, morning snacking was found to be less frequent among all age groups than in afternoon or evening snacking [27]. Using data from the 2009–2012 National Health and Nutrition Examination Survey (NHANES), Wang et al. showed that the afternoon period was the most reported snacking period among 4- to 13-year-old US children, followed by the evening and the morning periods [8]. In another study, it was found that almost half of the EDSF and one-third of the energy-dense drink (EDD) events in Dutch children aged 7–12 years occurred in the afternoon [17]. Therefore, afternoon and evening snacks are more frequent than morning snacking, especially among older age groups.

Among Canadian children and adolescents, a high proportion of “Vegetables and Fruit” (28–34%) and “Milk and Alternatives” (23–29%) food groups and lesser proportions of “Grain Products” and “Meat & Alternatives” food groups were consumed at snacking occasions. In addition, the contribution of snacking to “Other Foods & Beverages” ranged from 18% in younger children to 24% in adolescents. Using data from 2001 to 2004 NHANES, Sebastian and colleagues reported that snacking contributed to more than one-third of all fruit portions and oils, about one- fourth of all milk and grain portions, and lesser proportions of vegetables and meat/beans portions in adolescents aged 12–19 years. Additionally, over one-third of discretionary calories and added sugars came from snacking [28]. A study conducted in Spanish children aged 3–6 years and 7–12 years showed that fruit was the most commonly consumed food item among younger snackers (62%), followed by sandwiches, biscuits and yogurt. Among older snackers, fruit was the second most commonly consumed food item, after sandwich (64%) [29]. Wang et al. found that the most frequently consumed food group at snacks among 4- to 13-year-old US children were snacks and sweets, followed by nonalcoholic beverages, milk and dairy, and fruit [8]. Since taste preference is a major driver of an individual’s food selection [30, 31], improving the taste of healthy snacks, especially those consumed at home, may be beneficial in encouraging their consumption.

Among Canadian adults, a high proportion of “Milk and Alternatives” (22–24%) and “Vegetables and Fruit” (20–22%) food groups and lesser proportions of “Grain Products” and “Meat & Alternatives” were consumed at snacking occasions. In addition, the contribution of snacking to “Other Foods & Beverages” was significantly lower in older adults (16.4%) than adults (20.7%). The three most commonly consumed snacks among Brazilians (≥10 years) were sweetened coffee and tea (46.2%), sweets and desserts (31.5%), and fruit (31.1%) [32]. In another study conducted in Greek adults, the most frequently eaten food items as a snack were chocolates–cakes–ice cream, savoury pies and coffee [24]. Myhre et al. found that the top five energy-contributing snacks among Norwegian adults were cakes (334 kJ/day), fruits (213 kJ/day), sugar/sweets (186 kJ/day), bread and alcoholic beverages [14]. Results from the China Health and Nutrition Survey (CHNS) revealed that the top three contributors to snacking energy among Chinese adults were fruits, grains, and beverages [26].

### Study limitations

In this study, we used data from a recent nationally representative sample of Canadians to determine the time and location of snack consumption among different age groups. In addition, this survey used AMPM as a high-quality dietary assessment method. We also acknowledge some limitations to our study. First, dietary intake of participants were self-reported and was assessed using 24-h recall which may be subject to over and/or under reporting. Additionally, because our findings are only based on a single 24-h recall, the dietary intakes of each individual is not representative of their usual intake, although the values are representative at the population level. Secondly, the lack of a universally accepted definition of snacking may create some challenges in comparing the results across different studies, although we relied upon the participants to identify the eating occasions they considered as a snack, rather than imposing any time of day or calorie criteria [33, 34]. Third, the economic status and purchasing power may influence the patterns of snack consumption and the results may vary across different income groups.

### Study implications

This study has several implications for policy makers and researchers. We found that a large proportion of snacking events among Canadians occurs away from home. In brief, the propensity of adults to snack away from home highlights the opportunity for health promotion initiatives encouraging healthy food and drink availability outside their home, especially at work. Further, educating school-aged children and adolescents about healthy and safe snack foods and implementing healthy school food policies may promote healthy snacking behavior.

## Conclusions

Using comprehensive nutrition data from the 2015 CCHS-Nutrition, we determined the time, frequency and location of snack consumption among a representative sample of Canadians. Previously, we found that snacking is an important source of calories in the diet. In this study, we sought to better understanding snacking behaviors of Canadians and found that snacks are most commonly consumed at home, in the afternoon and evening, and that a high proportion of Milk and Alternatives and Vegetables and Fruit food groups are consumed at snacks. Determining the time and location of snack consumption is essential to a full understanding of snacking behavior in order to develop targeted strategies to improve the quality of snack food choices. Future research should attempt to understand how the time of day and location of snacking may influence the type and quality of snack food items, and how the snacking behavior may differ among Canadians from different socioeconomic classes.

## Data Availability

The data that had been used in this study is available at Statistics Canada Research Data Centres.
